# Subretinal Transplantation of Human Central Nervous System Stem Cells Stimulates Controlled Proliferation of Endogenous Retinal Pigment Epithelium

**DOI:** 10.1167/tvst.8.3.43

**Published:** 2019-06-19

**Authors:** Trevor J. McGill, Linda Osborne, Bin Lu, Jonathan Stoddard, Stephen Huhn, Ann Tsukamoto, Alexandra Capela

**Affiliations:** 1 Department of Ophthalmology, Casey Eye Institute, Oregon Health & Science University, Portland, OR, USA; 2 Division of Neuroscience, Oregon National Primate Research Center, Oregon Health & Science University, Beaverton, OR, USA; 3 StemCells, Inc., Newark, CA, USA; 4 Regenerative Medicine Institute, Cedars-Sinai Medical Center, Los Angeles, CA, USA; 5 Current address: BOCO Silicon Valley, Palo Alto, CA, USA

**Keywords:** cell transplantation, RPE, proliferation, neural stem cells, age related macular degeneration

## Abstract

**Purpose:**

The loss of retinal pigment epithelial (RPE) cells is a feature common to age-related macular degeneration (AMD) and retinitis pigmentosa (RP) and multiple early phase clinical trials are underway testing the safety of RPE cell replacement for these diseases. We examined whether transplantation of human neural stem cells into the subretinal space could enhance the endogenous proliferative capacity of the host RPE cell to regenerate.

**Methods:**

Human central nervous system stem cells (HuCNS-SC) were isolated from enzymatically treated brain tissue using flow cytometry. Pigmented dystrophic Royal College of Surgeons (RCS) and S334ter-4 rats treated with oral bromodeoxyuridine (BrdU) received a unilateral subretinal injection of 1.0 × 10^5^ HuCNS-SC cells at either postnatal day 21 or 60. Animals were sacrificed at 90, 120, and 150 days of age. Eyes were fixed processed for cryostat sectioning. Sections were immunostained with Stem101, Ku80, RPE65, OTX1/2, BrdU, and CRALBP antibodies and analyzed via confocal microscopy.

**Results:**

RCS rats that received transplantation of HuCNS-SC had significantly more (approximately 3-fold) Ki67-positive or BrdU-labelled host RPE cells adjacent to the HuCNS-SC graft than controls. Significantly increased host RPE cell proliferation as a result of HuCNS-SC transplantation also was confirmed in S334ter-line 4 transgenic rats with higher proliferation observed in animals with longer posttransplantation periods.

**Conclusions:**

These results suggest that controlled proliferation of endogenous RPE by HuCNS-SC may provide another mechanism by which RPE cell diseases could be treated.

**Translational Relevance:**

Engaging the capacity for endogenous RPE cell regeneration in atrophic diseases may be a novel therapeutic strategy for degenerative diseases of the RPE and retina.

## Introduction

The retinal pigmented epithelium (RPE) is a layer of highly polarized supportive cells that are critical for survival and function of the retina. The RPE is sandwiched between Bruch's membrane on the basal side and the neural retina on the apical side. Microvilli from the apical side of the RPE protrude into the photoreceptor layer and envelop photoreceptor outer segments (OS). This intimate connection between the RPE and photoreceptors enables the RPE to efficiently phagocytose shed OS, recycle oxidized photopigments, and provide additional critical trophic support to the outer retina while also removing waste.^1^,^2^ Thus, dysfunction or death of the RPE can have significant consequences for the survival of the host retina and ultimately for visual function.^3^ Indeed, millions of patients worldwide suffer from progressive and permanent vision loss due to RPE death/dysfunction in diseases, such as advanced age-related macular degeneration (AMD), Stargard's macular dystrophy, and certain forms of retinitis pigmentosa (RP).

Cell transplantation remains a promising experimental therapy for treatment of neurodegenerative diseases that affect the retina. In AMD and multiple forms of RP, photoreceptor degeneration occurs secondarily as a consequence of RPE degeneration or dysfunction. As a result, significant effort has been placed on transplantation of healthy RPE cells into animal models with dysfunctional RPE, thereby restoring or preventing secondary loss of host photoreceptors and their function.^4^,^5^ In the immunosuppressed Royal College of Surgeons (RCS) rat, a model of RPE cell dysfunction,^6^ transplantation of RPE cells before significant photoreceptor degeneration has occurred (at approximately postnatal day 21 or P21) and has shown measurable efficacy as confirmed by preservation of photoreceptors and behaviorally-measured visual acuity as well as reduction in the loss of electrophysiologic responses in the retina and brain.^7^–^25^ In general, transplanted RPE cells have been demonstrated to survive up to approximately 200 days and perform physiologic functions, such as ingestion of shed photoreceptor outer segments.^10^,^12^ While most studies of cell transplantation into the eye have focused on the rescue of vulnerable or dying photoreceptors, few have reported on interaction of the transplanted cells with the host RPE cell layer, and thus, the potential effects of cell transplantation on the host RPE cell layer itself have not been well investigated.

A particularly effective cell type used to prevent photoreceptor degeneration in the RCS rat is the human central nervous system stem cell (HuCNS-SC). Previous studies at our laboratory have shown that HuCNS-SC survive for at least 8 months after transplantation into the subretinal space of P21 RCS rats, where they preserved host photoreceptors from degeneration, and minimized loss of vision long-term.^11^ The mechanism of action for this benefit was multifactorial, but included the ability of HuCNS-SC cells to phagocytose photoreceptor outer segments,^9^ a function previously thought to be specific to RPE. A secondary analysis of the proliferative status of the HuCNS-SC graft revealed a marked presence of Ki67 expression in the host RPE cell layer within close proximity of transplanted HuCNS-SC (McGill et al.^11^ and in additional unpublished studies where transplants were performed at P60 rather than at P21). This remarkable finding suggests the possibility that increased proliferation of host RPE cells occurred in direct or indirect response to signals emanating directly from or secondary to the HuCNS-SC graft.

Relatively little data support low-level homeostatic proliferation of RPE in rodents and humans,^26^–^29^ with the majority of division in RPE cells occurring at the periphery near the ciliary body (where the RPE appears less dense). Understanding how to tap into and potentially augment this apparent regenerative ability in a safe and controlled way would have high therapeutic value for retinal disease where RPE loss (and subsequent central vision erosion) appears unstoppable, such as the case of advanced AMD with geographic atrophy (GA). Thus, our previous Ki67 expression observations prompted careful subsequent investigations of cell transplantation-induced RPE proliferation in the RCS rat and in a second unrelated model of RP, the S334ter-4 transgenic rat, a rhodopsin mutant.^30^ Using long-term continuous oral administration of the thymidine analogue bromodeoxyuridine (BrdU), we demonstrated that RPE proliferation is augmented preferentially in the RPE layer directly adjacent to the HuCNS-SC graft in both rat models, indicating that this phenomenon is directly related to the transplanted cells, is not model-specific, and appears independent of the status of the underlying RPE.

## Methods

All studies were performed in accordance with the Association for Research in Vision and Ophthalmology Animal Statement for the Use of Animals in Ophthalmic and Vision Research.

### Cell Preparation

HuCNS-SC were generated as described previously.^31^,^32^ Briefly, donated second trimester (16–20 gestation weeks) human brain tissue was enzymatically treated and labeled with CD133 and CD24 antibodies. The CD133^+^CD24^−/lo^ population was aseptically sorted using a BD Vantage flow cytometer (Becton Dickinson, Franklin Lakes, NJ). Sorted cells were cultured in suspension in a chemically defined, serum-free culture medium composed of X-VIVO 15 medium supplemented with N2, heparin, N-acetyl cysteine (NAC), basic fibroblast growth factor (FGF2), epidermal growth factor (EGF) and leukemia inhibitory factor (LIF) at a density of 1 × 10^5^ cells/mL. When neurosphere size reached 200 to 250 μm, cultures were passaged by collagenase treatment and replated in the same medium. Preparation of cells for subretinal transplantation was performed as described previously.^9^,^11^

### Study Design

Pigmented dystrophic RCS rats and S334ter-4 hemizygous rats received unilateral 2 μL subretinal injections of 1.0 × 10^5^ HuCNS-SC cells at P21 (RCS and S334ter-4) and an additional RCS rat group received the same treatment at P60; contralateral eyes served as nontransplanted (NT) or medium-injected controls (Table 1). A single subretinal HuCNS-SC injection was performed using a transscleral approach under general anesthesia as described previously.^10^,^11^,^15^ All animals were given intraperitoneal (i.p.) dexamethasone (DEX; 1.6 mg/kg) starting on the day of transplantation; rats transplanted at P21 were given DEX daily for 2 weeks while rats transplanted at P60 received DEX every 2 to 3 days (total of three injections); all rats also were maintained on oral cyclosporine A (210 mg/L) from 1 day before transplantation until the day of sacrifice. All rats in the study received a subcutaneous microchip with a unique 10-character identifier. Rat identification was retrieved using a transponder. Continuous oral administration of BrdU was achieved by dissolving the drug in the drinking water (in conjunction with the immunosuppressant Cyclosporine A) at a concentration of 1 mg/mL. Water containing BrdU and immunosuppressant drug was changed weekly and disposed of as hazardous waste according to the Alameda County regulations.

**Table 1:** Experimental Design – All Transplant Studies

Model	Age at Transplant	Marker Analyzed	Number of Eyes	Age at Sacrifice	Transplant Duration (days)
RCS	P21	Ki67	*n* = 6 (cells)	P90	∼70
			*n* = 7 (medium)		
RCS	P60	Ki67	*n* = 3 (cells)	P90	∼30
			*n* = 3 (NT)		
			*n* = 5 (cells)	P120	∼60
			*n* = 3 (NT)		
RCS	P60	BrdU	*n* = 7 (cells)	P120	∼60
			*n* = 5 (medium)		
			*n* = 4 (NT)		
S334ter-4	P21	BrdU	*n* = 3 (cells)	P90	∼70
			*n* = 2 (medium)		
			*n* = 2 (NT)		
			*n* = 3 (cells)	P150	∼130
			*n* = 2 (medium)		
			*n* = 2 (NT)		

### Histology of Transplanted Retinas

All animals were sacrificed by CO2 inhalation followed by perfusion with phosphate-buffered saline (PBS). RCS rats were sacrificed at P90 and P120 (∼30 and 60 days after transplantation while the S334ter-4 rats were sacrificed at P90 and P150 (∼70 and 130 days after transplantation). The eyes were removed and immersion fixed in 2% paraformaldehyde for 1 hour, followed by cryopreservation in sucrose and embedding in optimum cutting temperature (OCT) compound. Horizontal sections (10 μm) were cut on a cryostat and every 10th slide was stained with cresyl violet for assessment of injection site, donor cell engraftment, and migration as well as photoreceptor preservation. Sections were immunostained with various antibodies as follows: mouse monoclonal anti-Stem101 (1:1000; Takara Bio, Kusatsu, Japan), rabbit anti-Ku80 (1:250; Abcam, Cambridge, UK), mouse anti-RPE65 (1:250; Abcam), rabbit anti-OTX1/2 (1:250; Abcam), rabbit anti-Ki67 (1:400; Abcam), rat anti-BrdU (1:250; Serotec, Kidlington, UK), mouse anti-BrdU (1:250; BD Biosciences, Billerica, MA), mouse anti-CRALBP (1:200; Abcam). Secondary antibodies used were donkey anti-mouse Alexa 488 and donkey anti-rabbit Alexa 568 (Invitrogen, Carlsbad, CA), donkey F(ab)2 anti-rat Cy3 and donkey anti-mouse Dylight 649 (Jackson Immunoresearch Laboratories, West Grove, PA), all used at 1:500 dilution. Counterstaining was achieved using DAPI (1:1000; Invitrogen). BrdU staining was the last step of any double/triple staining protocol; sections were incubated in 2M hydrochloric acid for 30 minutes at 37°C before incubation with the chosen BrdU primary antibody made in rat or mouse, depending on the staining combination (in double stainings with primary antibodies made in mouse, such as RPE65 or CRALBP, the rat BrdU was used).

### Imaging and Quantification

Fluorescence staining was analyzed by fluorescence and confocal microscopy. Select images were filter and/or color intensity corrected (Volocity 6.3; PerkinElmer, Waltham, MA) for publication purposes – no other image manipulation was conducted. The number of Ki67^+^RPE65^+^ cells and BrdU^+^RPE65^+^ (or BrdU^+^OTX1/2^+^) RPE cells were quantified in the following manner: in NT and medium transplanted eyes, fluorescently-labeled double-positive cells were quantified by direct examination in four adjacent, nonoverlapping temporal fields of 300 μm length (total length per retina section was 1200 μm); the first quantification field was considered after a two-field guard to avoid sampling from the most peripheral RPE adjacent to the ciliary epithelium, an area known to contain proliferative RPE in normal rats and mice.^26^,^27^ A total of four to six slides per eye were examined, corresponding to a maximum of 24 retina sections. In HuCNS-SC transplanted eyes, adjacent, nonoverlapping confocal images (375 μm) were taken of the RPE layer adjacent to the HuCNS-SC graft. As with control eyes, the most peripheral RPE was avoided. Interestingly, HuCNS-SC were rarely found near the periphery, so our sampling method naturally avoided those areas. Results were expressed as either the total number of Ki67^+^RPE65^+^ cells per retina section or the total number of BrdU^+^ RPE cells (OTX1/2^+^ or RPE65^+^) per 100 μm retina length. Note that in previous studies we had used this type of quantification to determine survival of cones in transplanted, medium and NT retinas.^11^

### Statistics

A linear mixed model was used to calculate statistical significance of group effects across ages and groups. Where group effects were significant (*P* < 0.05), subsequent 1-way analysis of variance (ANOVA) with Bonferroni post hoc analysis or 2-way *t*-tests also were performed.

## Results

### RPE Cells Adjacent to the HuCNS-SC Graft Express the Proliferation Marker Ki67

**Figure 1:**
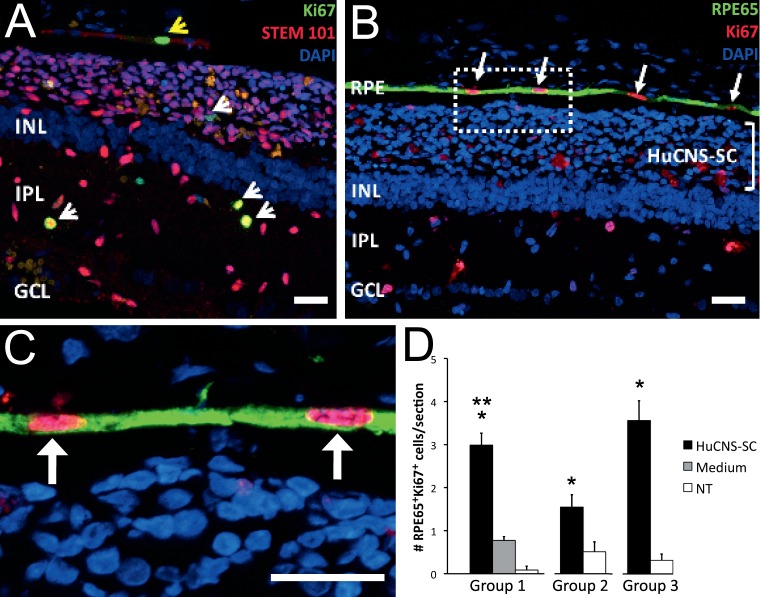
RPE cells proliferate in HuCNS-SC-transplanted RCS rats. (A) Representative image of a P60->P120 RCS rat section showing that a small percentage of HuCNS-SC (STEM101^+^, red) located in the subretinal space or in the INL, express Ki67 (yellow nuclear stain, white arrowheads). The yellow arrowhead points to a Ki67^+^ non-HuCNS-SC cell located in the RPE layer. (B) Confirmation that RPE cells, identified by their expression of RPE65 (green), can also express Ki67 (red, white arrows). The subretinal HuCNS-SC graft is bracketed. The boxed area in (B) is shown as a higher magnification insert in (C). (C) The white arrows point to two adjacent Ki67^+^ (red) RPE65^+^ (green) RPE cells. (A–C) Nuclei stained with 4′,6-diamidino-2-phenylendole (DAPI). Scale bar: 25 μm. INL, inner nuclear layer; IPL, inner plexiform layer; GCL, ganglion cell layer. (D) Quantification of the number of RPE65^+^Ki67^+^ cells per retina section in RCS rats transplanted at P21 and P60. Results are shown as average ± SEM. Group 1: animals injected on P21 and histology analyzed on P90. Single asterisk (*) indicates significant difference between HuCNS-SC and medium (P < 0.001) and double asterisk (**) indicates significant difference between HuCNS-SC and NT (P < 0.001). Group 2: animals injected on P60 and histology analyzed on P90. Single asterisk (*) indicates significant difference between HuCNS-SC and NT (P = 0.024). Group 3: animals injected on P60 and histology analyzed on P120. Single asterisk (*) indicates significant difference between HuCNS-SC and NT (P = 0.0063).

Our previous work describing long-term efficacy of HuCNS-SC transplantation in the RCS rat also illustrated the presence of a small subpopulation of proliferative HuCNS-SC (<7%) throughout the life of the graft (up to 210 days, the longest time point tested), based on Ki67 staining.^11^ In that same group of animals, Ki67 expression also was detected in the host RPE cell layer immediately adjacent to the human cell graft. Interestingly, those Ki67^+^ cells coexpressed RPE65, a specific marker of RPE cells, but not the STEM101 human-specific marker, suggesting the proliferative cells were host RPE and not HuCNS-SC cells embedded in the RPE layer. Additionally, in all our transplantation studies to date in which HuCNS-SC were injected into the subretinal space, we never observed donor cells engraft in the RPE layer nor to express RPE65.^9^,^11^ Because RPE cells are thought to be mostly postmitotic, we decided to further investigate this initial observation and quantified the number of RPE65^+^Ki67^+^ cells in transplanted and control eyes of RCS rats injected and sacrificed at different time points. We found that in RCS rats transplanted with HuCNS-SC at P21 and examined at P90 (approximately 70 days after transplantation), an average of 2.99 ± 0.28 RPE65^+^Ki67^+^ cells per retina section were detected (Fig. 1D, group 1); however, only 0.76 ± 0.09 and 0.08 ± 0.08 RPE65^+^Ki67^+^ cells were found in medium transplanted and NT eyes, respectively, suggesting that the HuCNS-SC graft may stimulate proliferation in RPE cells by a factor of approximately 3 over this time period. Interestingly, all RPE65^+^Ki67^+^ cells in transplanted retinas were found adjacent to the HuCNS-SC graft, indicating that the close proximity between the graft and RPE layer was a potential driver for this phenomenon.

We then examined RCS rats transplanted at P60, an age when the underlying disease is more advanced and evidence of efficacy could potentially be more meaningful from a translational perspective. We found that in animals sacrificed at P90 or P120 (30 or 60 days after transplantation), RPE65^+^Ki67^+^ cells also were present in the RPE layer adjacent to HuCNS-SC graft (Figs. 1A–C) and, as in the case of RCS rats transplanted at P21, these cells were not STEM101^+^ human cells. We found an average of 1.55 ± 0.29 RPE65^+^Ki67^+^ cells in cell-treated eyes versus 0.50 ± 0.22 RPE65^+^Ki67^+^ cells in NT eyes at P90 (Fig. 1D, group 2). At P120, 60 days after injection, we found this trend increased to 3.56 ± 0.46 RPE65^+^Ki67^+^ cells in cell-treated eyes versus 0.31 ± 0.15 RPE65^+^Ki67^+^ cells in NT eyes (Fig. 1D, group 3). The data at P60 show a difference in the number of proliferating cells in transplanted versus NT eyes by factors of 3- and 10-fold, respectively. In addition, the number of RPE65^+^Ki67^+^ cells in the P21 to P90 (2.99 ± 0.28) and P60 to P120 (3.56 ± 0.46) groups were quite similar, and both had similar posttransplantation times. Furthermore, the increase in the number of RPE65^+^Ki67^+^ cells in cell-treated eyes at P120 compared to P90 suggested that the apparent stimulatory effect of HuCNS-SC on RPE proliferation is present throughout the posttransplant time.

### BrdU Uptake by RPE Cells in the RCS Rat is Stimulated by the HuCNS-SC Graft

**Figure 2:**
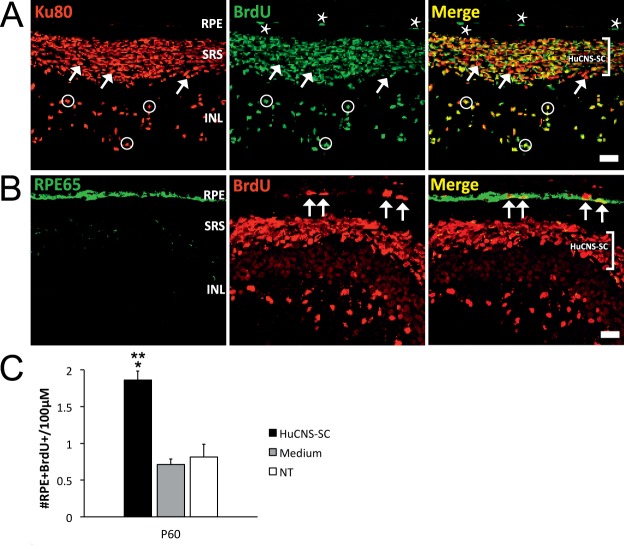
RPE cells incorporate BrdU in RCS rats transplanted at P60 with HuCNS-SC. (A) HuCNS-SC, labeled with the Ku80 nuclear marker (red) can be found in the subretinal space (SRS; white bracket) and also in the INL, a consequence of inner retinal migration. BrdU^+^ (green) cells are abundant in the SRS and also in the inner retina. Numerous Ku80^+^BrdU^+^ cells also are present in the SRS and in the INL (white circles), indicating a relatively high proliferative capacity of the HuCNS-SC. White arrows highlight some examples of Ku80^+^ donor cells that do not express BrdU. More importantly, BrdU expression is detected in Ku80^−^ cells located in the RPE layer (white asterisks). (B) BrdU expression (red, middle panel), in the RPE layer adjacent to a SRS HuCNS-SC graft (white bracket). BrdU^+^RPE65^+^ RPE cells (white arrows at middle and right) appear yellow in the merged image (although RPE65 and BrdU expression are in separate cellular compartments, the image is a flattened composite of approximately 20 pictures taken in individual confocal planes; therefore, giving the impression of colocalization). Scale bar: in (A) and (B) 37μm. (C) HuCNS-SC transplanted eyes (HuCNS-SC) have significantly more proliferative RPE cells than NT and vehicle-injected (medium) eyes. NT (n = 4); Medium (n = 5), and HuCNS-SC (n = 7). Results are shown as average ± SEM. There was a main effect of treatment (P < 0.001); post hoc analysis indicated a significant difference between HuCNS-SC–treated eyes and medium-injected eyes (*P < 0.0001), and a significant difference between cells and NT eyes (**P = 0.0002). There was no statistical difference between media and NT eyes.

Antibodies against Ki67 reveal positive staining if cells are in the G1, S, or G2 phases and negative in the G0 phase; therefore, Ki67 staining only provides a snapshot of the potential number of proliferating cells (albeit in different phases of the cell cycle) at sacrifice. To label all cells that actually go through DNA replication (or S phase) within a given time interval, we repeated the subretinal HuCNS-SC transplantation experiment while administering BrdU in the drinking water during the entire after transplantation period (Table 1). The maximum number of RPE65^+^Ki67^+^ cells observed in the studies described above occurred in RCS rats transplanted at P60 and sacrificed at P120, and, therefore, for consistency and comparative purposes, we decided to inject HuCNS-SC at P60 and terminate all animals given BrdU at P120. In all study eyes (transplanted, medium-injected and NT), we observed BrdU incorporation within the RPE layer (characterized by RPE65 or Otx1/2 expression), particularly noticeable near the ciliary body (not shown); BrdU^+^ cells also were present in host ocular tissues including the choroid and within the neural retina. Staining with anti-BrdU and Ku80 antibodies in transplanted eyes revealed the presence of numerous BrdU-positive cells within the HuCNS-SC graft in the subretinal space, indicating that the vast majority of donor cells proliferated during the life of the graft (Fig. 2A). More interestingly, and similarly to the Ki67 data presented above, we found numerous examples of BrdU-positive RPE cells adjacent to the HuCNS-SC graft (Fig. 2B). Quantification of labeled cells (Fig. 2C) indicated that there were 1.86 ± 0.121 BrdU^+^RPE cells/100 μm retina length in areas of HuCNS-SC engraftment whereas similar temporal areas in medium-injected and NT control eyes had averages of 0.71 ± 0.075 and 0.82 ± 0.171 BrdU^+^RPE cells/100 μm retina length, respectively. In nasal area of transplanted eyes, the average number of BrdU^+^RPE cells was negligible (not shown). These data are in agreement with the observations obtained with Ki67 staining and appear to support the hypothesis that HuCNS-SC engraftment stimulates proliferation of RPE cells in the RCS rat.

### Proliferative RPE Cells Express Markers of Bona Fide RPE

**Figure 3:**
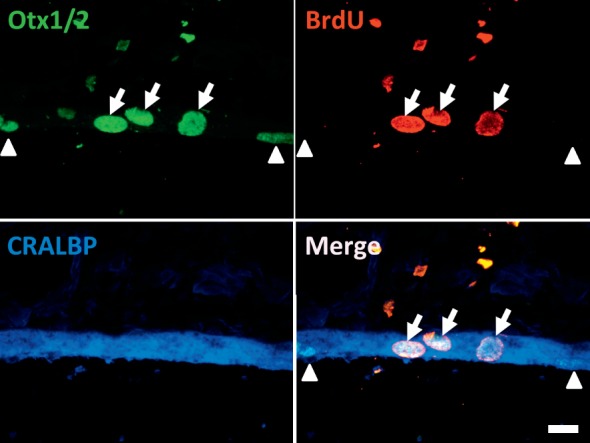
BrdU^+^ cells in the RPE layer are bona fide RPE cells. Representative image of BrdU expression in the RPE layer showing that BrdU^+^Otx1/2^+^ RPE cells also express CRALBP (white arrows), indicating that the newborn RPE cells exhibit expression of key RPE markers. Two nonproliferative Otx1/2^+^CRALBP^+^ RPE cells (BrdU^−^) also are shown (white arrowheads). Scale bar: 12 μm.

An important question that arises from this study is whether the newly born RPE cells (which incorporate BrdU) are bona fide RPE cells. Based on our staining results, all the BrdU^+^ cells present in the RPE layer also were Otx1/2 and/or RPE65-positive, suggesting that indeed these cells are RPE cells. We furthered this analysis by also staining retinas with the cellular retinaldehyde-binding protein (CRALBP) marker; like RPE65, CRALBP is a key visual cycle protein expressed in RPE and also in Müller glia. In RPE cells, CRALBP is involved in the intracellular transport of hydrophobic 11-cis retinoids. We found that all BrdU^+^Otx1/2^+^ cells also were CRALBP-positive, further confirming the expression of this key protein in newly born RPE cells (Fig. 3). Based on the coexpression of the standard RPE markers Otx1/2 and CRALBP in BrdU^+^ cells, we concluded that these are bona fide host RPE cells.

### Subretinal HuCNS-SC Graft Stimulate RPE Proliferation in the S334ter-4 Transgenic Rat

The RCS rat has a mutation in the *mertk* gene that impairs the RPE's normal ability to phagocytose photoreceptor outer segments,^6^ and as a result, the overlaying photoreceptors die in a progressive and predictable fashion. Arguably then, the dysfunctional RPE in the RCS rat may not represent the typical response of normal RPE as seen in multiple retinal diseases where the primary mutation is located extra-RPE, typically within photoreceptors. Thus, to determine whether HuCNS-SC-stimulated RPE proliferation was model-specific, we elected to repeat some of the aforementioned experiments in a different RP model. For this purpose, we transplanted HuCNS-SC into S334ter-4 rats at P21 and sacrificed the animals at P90 (∼70 day posttransplantation assessment) and also at P150 (∼130 days after transplantation). The time point P90 provided a direct comparison to the initial study in the RCS rat (P21–90), as well as a comparable posttransplantation cell survival to the P60 injected animals (P60–P120). Addition of the P150 time point allowed us to determine whether, similarly to what we observed in RCS rats transplanted at P60, there was any change in the number of proliferative RPE cells with postengraftment time. All animals in this study were provided BrdU from the day of injection through sacrifice.

**Figure 4:**
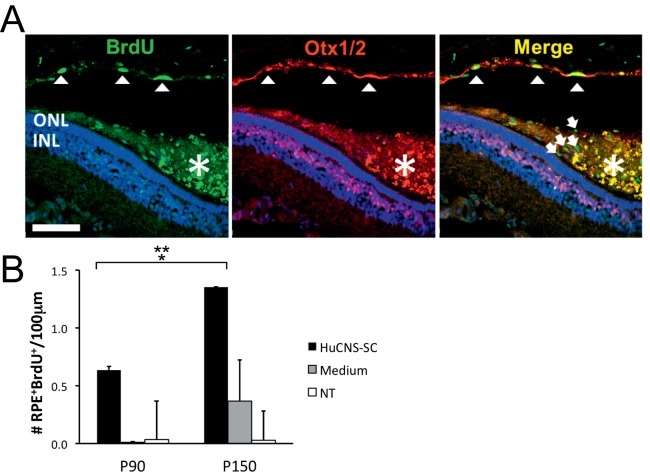
RPE cells incorporate BrdU in S334ter-4 rats transplanted at P21 with HuCNS-SC. (A) Representative images of BrdU expression (green) in the RPE layer (Otx1/2^+^, red) adjacent to a HuCNS-SC graft (white asterisk) at P90. Three BrdU^+^Otx1/2^+^ RPE cells are marked with white arrowheads. BrdU^+^ cells within the HuCNS-SC graft are noted by white arrows in the merge image. Of note is the relatively high autofluorescence in the red and green channel (appear yellow in the merge image) the graft area. Scale bar: 70 μm. (B) HuCNS-SC transplanted eyes (HuCNS-SC) have more proliferative RPE cells than NT and medium-injected (medium) eyes. NT (n = 2); Medium (n = 2) for each posttransplant time point; HuCNS-SC (n = 3, P90; n = 3, P150). Results are shown as average ± SEM. There was an overall effect of treatment group (P = 0.011). *Significant difference between cells and medium treated (P = 0.015). **Significant difference between cells and untreated (P = 0.006). There was no difference between medium and NT groups.

Staining of S334ter-4 eye sections with anti-BrdU and Otx1/2 antibodies revealed the presence of numerous BrdU^+^ cells in the HuCNS-SC graft (Fig. 4A, white arrows in merge panel), similar to that observed in the RCS rat (Fig. 2). Transplanted eyes also exhibited a number of cells in the RPE layer adjacent to the HuCNS-SC graft that were BrdU^+^ (Fig. 4A, white arrowheads). Quantification of the number of proliferative RPE cells (Fig. 4B) at P90 showed that there were 0.63 ± 0.033 BrdU^+^Otx1/2^+^ cells/100 μm retina length in areas of HuCNS-SC engraftment whereas similar temporal areas in medium-injected and NT control eyes had averages of 0.01 ± 0.005 and 0.03 ± 0.033 BrdU^+^Otx1/2^+^ cells/100 μm retina length, respectively. At P150, the cell- and medium-injected eyes exhibited an increase in the number of BrdU^+^Otx1/2^+^ cells/100 μm retina length to 1.35 ± 0.253 and 0.37 ± 0.355 respectfully, while NT eyes maintained a similar number to that reported for P90, 0.03 ± 0.033. These data indicated that endogenous proliferation of RPE cells is a rare event in control eyes regardless of posttransplant time. We found that at P150 there were more BrdU^+^Otx1/2^+^ cells than at P90 in HuCNS-SC-injected eyes, indicating that, similarly to what we found in the RCS rats transplanted at P60, HuCNS-SC-stimulated RPE proliferation in the S334ter-4 rat is likely a continuous process after transplantation, and not merely an acute response to HuCNS-SC transplantation. Finally, the level of RPE proliferation was similar in the RCS and the S334ter-4 rats (compare Fig. 2C to Fig. 4). Taken together, the data obtained in two distinct rat RP models suggested that a controlled RPE proliferative response to a HuCNS-SC transplant may be a general characteristic with important translational value.

## Discussion

Our results demonstrated that endogenous RPE cells of RCS and S334ter-4 rat models: (1) inherently proliferate at very low levels, (2) this proliferation can be modestly stimulated by surgical manipulation of the retina, (3) the presence of a subretinal HuCNS-SC graft significantly increases the host RPE cells proliferative capacity, and (4) increased postinjection duration results in increased proliferation of the host RPE. To our knowledge, this is the first study that demonstrates host RPE cell proliferation in response to any type of donor cell transplantation.

Previous studies in rats and mice without retinal degeneration have shown that a relatively small number mature RPE cells proliferate, particularly those residing in the peripheral retina.^26^,^27^ The fact that we observed increased RPE cell proliferation in models with dysfunctional (RCS) as well as normal (S334ter-4) RPE is important as it suggests that under the proper circumstances, RPE cells have the ability to reenter the cell cycle and that this ability may be independent of the underlying health status of the RPE layer. These findings could have significant clinical value as they point towards a potential treatment strategy for retinal degenerative diseases, such as AMD and the majority of cases of RP where RPE cells die but are not inherently dysfunctional. With a mechanism of action that combines host RPE replenishment with photoreceptor neuroprotection, HuCNS-SC could be viewed as a two-pronged therapeutic strategy for the aforementioned diseases and underscores the value of continued clinical development of these cells for diseases, such as macular degeneration.

While repopulation of the host RPE cell layer could potentially hold significant therapeutic value, it remains unclear whether there are functional or safety consequences of RPE proliferation on the normal functioning of the RPE layer and/or the physiology of adjacent photoreceptors. From a safety perspective, it should be noted that RPE proliferation was augmented only in the vicinity of the HuCNS-SC graft, suggesting that the signal responsible for this RPE response acts over a limited microscopic distance. Furthermore, not all RPE cells adjacent to the HuCNS-SC graft were Ki67^+^ or BrdU^+^; in fact, the number of BrdU^+^RPE cells was relatively low. This suggests that only a subset of the RPE population retains the ability to proliferate, and/or that, in the absence of obvious RPE loss, there is a maximum proliferative capacity, likely constrained by space or mechanisms that prevent uncontrolled RPE proliferation.^29^ In this and our previous studies,^9^,^11^ we did not observe any functional or anatomical abnormalities as a result of the controlled RPE proliferation. In fact, the results of our efficacy studies in the RCS rat indicated that transplantation of HuCNS-SC rescued photoreceptors from degeneration, rescued electrophysiologic responses of the visual system, rescued behaviorally measured vision, and that the cells were able to phagocytose photoreceptor outer segments. However, in the absence of the transplanted cells, the retina and visual function degenerated in a progressive and predictable fashion as described previously.^8^,^33^–^35^ In addition, neither IND-enabling GLP safety and toxicology studies that supported the Phase I/II clinical trial (NCT01632527) nor the safety data for the trial itself (manuscript in preparation) showed evidence of uncontrolled RPE proliferation reminiscent of proliferative vitreoretinopathy (PVR) in the area of the HuCNS-SC graft or elsewhere in the treated eye.

The observation that not all RPE cells responded to the signals provided by the graft seems to point towards the existence of a subpopulation of RPE cells that is poised to reenter the cell cycle if the conditions are met. To our knowledge no studies address this possibility and/or describe markers that subdivide RPE cells from a functional perspective. Two previous studies have indicated that RPE cells isolated from human eyes can proliferate in vitro in response to the growth factor FGF2, but it is unclear from their study whether all cells have this capacity or only a subpopulation of RPE.^36^,^37^ A previous report using albino rats described that RPE proliferation is more extensive than in normal pigmented rats, and the investigators speculated that there might be an inverse relationship between photoreceptor numbers (and their contact with RPE), and the probability of RPE cells entering the cell cycle.^26^ It is possible that the neurodegenerative environment of photoreceptor loss in the RCS and S334ter-4 rats could provide an initial signal for RPE proliferation, which then could be further stimulated by cytokines or growth factors released by the HuCNS-SC graft. In the absence of markers that could identify proliferation-ready RPE cells, we can only speculate their existence as part of a dormant regenerative program that could be awaken by photoreceptor loss and effectively stimulated by a specific therapy.

The two rodent models used in this study are commonly used to evaluate prospective therapies for retinal degenerative disease. Albeit retinal degeneration occurs through different primary mechanisms,^38^,^39^ both models undergo photoreceptor degeneration but neither exhibits clear RPE loss. In the absence of clear endogenous RPE cell loss, the increase in proliferation generates new bona fide RPE cells that appear to incorporate seamlessly in the RPE monolayer, as evidenced by the lack of anatomic disturbances noted in all of our HuCNS-SC transplantation studies to date. An investigation of the consequences of HuCNS-SC transplantation on RPE proliferation in models with progressive RPE cell loss is of particularly high relevance and is the subject of ongoing and future studies. Finally, our study did not address the mechanism by which HuCNS-SC stimulate RPE proliferation. Our working hypothesis is that HuCNS-SC may release known RPE mitogens, such as FGF2, EGF, hepatocyte growth factor (HGF) and platelet derived growth factor (PDGF).^40^ Indeed, our own unpublished microarray data indicated that ex vivo expanded HuCNS-SC abundantly express the transcripts for those genes. Transplantation of HuCNS-SC engineered to express reduced levels of these key mitogens could potentially shed light into the mechanism of action underlying our observations.

It has been proposed that peripherally generated new RPE cells could potentially replenish RPE cell loss that occurs centrally through normal aging in humans^28^ or due to degenerative processes, such as AMD with GA.^41^ Our data in this study suggested a role for HuCNS-SC in augmenting that putative regenerative capacity, particularly in the context of AMD with GA. From an efficacy perspective, the level of RPE proliferation that could give rise to a meaningful clinical benefit remains unknown. Provocatively, the results of the Phase I/II study in AMD-GA have shown a trend for a decrease in the total area of GA progression in the eye that received a single perimacular subretinal injection of HuCNS-SC versus the untreated fellow eye (manuscript in preparation). This result is consistent with the potential effect of transplantation on the underlying human RPE, either through metabolic support to the host RPE cells (healthy and diseased) and/or possibly through stimulation of RPE regeneration as described in the preclinical rat models. The possibility that HuCNS-SC may provide a meaningful impact on the biology of human RPE in AMD-GA, in addition to neuroprotective effects on the photoreceptors, is extremely intriguing as it indicates that intervention with HuCNS-SC is likely to provide multimechanistic therapeutic benefit in retinal degenerative diseases.
